# CT perfusion-based delta-radiomics models to identify collateral vessel formation after revascularization in patients with moyamoya disease

**DOI:** 10.3389/fnins.2022.974096

**Published:** 2022-08-11

**Authors:** Jizhen Li, Yan Zhang, Di Yin, Hui Shang, Kejian Li, Tianyu Jiao, Caiyun Fang, Yi Cui, Ming Liu, Jun Pan, Qingshi Zeng

**Affiliations:** ^1^Department of Radiology, Shandong Provincial Qianfoshan Hospital, Shandong University, Jinan, China; ^2^Department of Radiology, Shandong Mental Health Center Affiliated to Shandong University, Jinan, China; ^3^Department of Radiology, The First Affiliated Hospital of Shandong First Medical University, Shandong Provincial Qianfoshan Hospital, Jinan, China; ^4^Department of Radiology, Qilu Hospital of Shandong University, Jinan, China; ^5^Department of Neurosurgery, Qilu Hospital of Shandong University, Jinan, China

**Keywords:** perfusion imaging, moyamoya disease, cerebral revascularization, delta-radiomics, machine learning

## Abstract

**Purpose:**

To build CT perfusion (CTP)-based delta-radiomics models to identify collateral vessel formation after revascularization in patients with moyamoya disease (MMD).

**Methods:**

Fifty-three MMD patients who underwent CTP and digital subtraction angiography (DSA) examination were retrospectively enrolled. Patients were divided into good and poor groups based on postoperative DSA. CTP parameters, such as mean transit time (MTT), time to drain (TTD), time to maximal plasma concentration (Tmax), and flow extraction product (FE), were obtained. CTP efficacy in evaluating surgical treatment were compared between the good and poor groups. The changes in the relative CTP parameters (ΔrMTT, ΔrTTD, ΔrTmax, and ΔrFE) were calculated to evaluate the differences between pre- and postoperative CTP values. CTP parameters were selected to build delta-radiomics models for identifying collateral vessel formation. The identification performance of machine learning classifiers was assessed using area under the receiver operating characteristic curve (AUC).

**Results:**

Of the 53 patients, 36 (67.9%) and 17 (32.1%) were divided into the good and poor groups, respectively. The postoperative changes of ΔrMTT, ΔrTTD, ΔrTmax, and ΔrFE in the good group were significantly better than the poor group (*p* < 0.05). Among all CTP parameters in the perfusion improvement evaluation, the ΔrTTD had the largest AUC (0.873). Eleven features were selected from the TTD parameter to build the delta-radiomics model. The classifiers of the support vector machine and k-nearest neighbors showed good diagnostic performance with AUC values of 0.933 and 0.867, respectively.

**Conclusion:**

The TTD-based delta-radiomics model has the potential to identify collateral vessel formation after the operation.

## Introduction

Moyamoya disease (MMD), also known as spontaneous occlusion of the circle of Willis, is a non-atherosclerotic progressive steno-occlusive arteriopathy first reported by Suzuki and Takaku (1969). It most frequently affects the internal carotid arteries, proximal segments of the middle cerebral arteries (MCAs), and anterior cerebral arteries, accompanied by a tuft of collateral vessels at the base of the brain. Revascularization could mitigate the risk of future ischemic events or MMD rebleeding (Miyamoto et al., 2014; Kim et al., 2016b). Thus, surgical interventions are recommended once the MMD diagnosis is clear (Narisawa et al., 2009; Shi et al., 2021). Revascularization can immediately improve cerebral blood flow. Superficial temporal artery–MCA anastomosis is most commonly used in clinical practice (Kim et al., 2016a; Acker et al., 2018).

Perfusion imaging provides an assessment of the territory at risk for infarct from hypoperfusion and serves a key role in making surgical decision for MMD. CT perfusion (CTP), as a fast, feasible, and multiparameter imaging modality, has been widely used in cerebral hemodynamic evaluation of MMD (Chen et al., 2016; Li et al., 2019; Guo et al., 2021). After revascularization, digital subtraction angiography (DSA) is the reference standard for evaluating the patency of the bypass and collateral vessel formation (Hwang et al., 2020). However, limited by multiple factors (e.g., invasive nature, radiation exposure, and perioperative complications), DSA is sometimes given more careful consideration (Bendszus et al., 1999). In recent years, artificial intelligence with radiomics as the core has made breakthroughs in computer-aided diagnosis, staging, and prognosis of diseases (Huang et al., 2016; Elhalawani et al., 2018; Gu et al., 2019; Zhang et al., 2020). Radiomics has the advantages of intelligence, multiple parameters, and objective quantification (Gillies et al., 2016). Delta-radiomics introduces a time component and shows the changes in radiomics features from pre- to post-treatment and is suitable for evaluating the treatment response (Lambin et al., 2017).

Studies on automated detection of MMD based on machine learning have been recently noted (Kim et al., 2019; Akiyama et al., 2020; Waddle et al., 2020; Lei et al., 2021). However, no published studies have focused on radiomics in predicting MMD treatment outcomes after revascularization. Therefore, this study aims to (1) evaluate the CTP efficacy to assess the perfusion changes before and after revascularization and (2) further build the delta-radiomics models to identify the formation of collateral vessels after the operation.

## Materials and methods

### Patients

**FIGURE 1:**
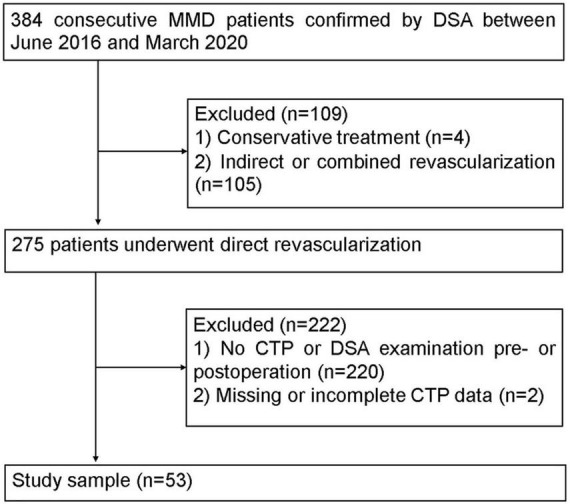
Flowchart of the study of the enrolled patients.

This retrospective study was approved by the local institutional review board, and the requirement for informed consent was waived. The MMD patients, based on the diagnostic guidelines of the Research Committee of MMD of the Japanese Ministry of Health (Research Committee on the Pathology and Treatment of Spontaneous Occlusion of the Circle of Willis and Health Labour Sciences Research Grant for Research on Measures for Infractable Diseases, 2012), were enrolled from June 2016 to March 2020 (Figure 1). The participation eligibility was established following the inclusion criteria: (1) the CTP and DSA were performed before and after surgery, (2) the surgical method was direct revascularization surgery. The exclusion criteria were as follows: (1) the hemisphere had undergone other surgery before revascularization and (2) the CTP data was incomplete or missing. After revascularization, DSA and CTP examinations were scheduled for patients at 6 months follow-up.

### Angiographic evaluation

The angiographic stages of the MMD patients were evaluated by DSA. All images were assessed by two radiologists (JL and YZ, with 8 and 10 years of experience, respectively). The collateral vessels were classified into four grades according to the extent of collateral vessel formation by modification of the Matsushima grade (Matsushima et al., 1992) (grade 0, no clear collaterals; grades 1, less than one-third of the MCA distribution; grade 2, between two-thirds and one-third of the MCA distribution; and grade 3, more than two-thirds of the MCA distribution). Grades 2 and 3 were classified as the good group, and grades 1 and 0 were classified as the poor group.

### CT scanning and processing of CT perfusion data

The CTP was performed using the third-generation dual-source CT scanner (Somatom Force, Siemens Healthcare, Forchheim, Germany). Routine brain scanning was done, and DynMulti 4D scan mode (shuttle scan mode, Siemens Healthcare) was used for volume perfusion computer tomography. A 50-ml bolus of contrast media iopromide (Ultravist 370 mg L/ml; Bayer Schering Pharma, Berlin, Germany) was administered into an antecubital vein using a power injector (Ulrich Injection System, Germany) with an injection rate of 5 ml/s. Eighteen dynamic CT scans were initiated 5 s after the start of the contrast material injection. The scanning parameters were as follows: 70 kV tube voltage, 200 mA tube current, collimator 128 mm × 0.6 mm, 1.5 mm slice thickness, and 0.25 s rotation time.

CT perfusion source data were transferred to the Syngo workstation (Siemens Syngo.*via*, VA20A). The data source was analyzed using CT Neuro-Perfusion software. The cerebral artery that first reached the enhanced peak was selected as the input artery. The superior sagittal sinus was selected as the output vein. Perfusion parameter maps were generated *via* an automatic delay-insensitive deconvolution algorithm (Abels et al., 2010). Parameter maps for cerebral blood flow (CBF), cerebral blood volume (CBV), time to drain (TTD), mean transit time (MTT), time to maximal plasma concentration (Tmax), and flow extraction product (FE) were obtained.

For quantitative analysis, the Tmax > 6 s was used as the threshold to define the ischemic hypoperfusion area (Zaro-Weber et al., 2019). Regions of interest (ROIs) were drawn in the largest cross-sectional area of the abnormal perfusion. The range of ROIs covered the complete perfusion abnormalities over the cortical MCA distribution. Previous hemorrhage and infarcted lesions were avoided in the ROI. Contralateral mirror ROIs were automatically acquired. The average value was taken after two measurements. The relative CTP values (e.g., rCBF, rCBV, rMTT, rTTD, rTmax, and rFE) were defined as the ratios between absolute CTP values of the surgical and contralateral sides. An attempt was made to draw the same ROIs in the same location for one patient before and after the revascularization as shown in a previous study (Kang et al., 2020).

To compare variation between the preoperative and postoperative perfusion parameters, the relative CTP parameter changes were calculated as follows: ΔrCBF = rCBFpost - rCBFpre, ΔrCBV = rCBVpost - rCBVpre, ΔrMTT = rMTTpre - rMTTpost, ΔrTTD = rTTDpre - rTTDpost, ΔrTmax = rTmaxpre - rTmaxpost, ΔrFE = rFEpre - rFEpost, where Δ, pre, and post indicate CTP parameter change, before surgery, and after surgery, respectively.

### Radiomics workflow

The workflow of the delta-radiomics analysis included ROI segmentation, feature extraction, feature reduction and selection, and model construction.

### ROIs segmentation

Two experienced radiologists (with 7 and 15 years of CT diagnosis experience) performed the segmentation using the open-source software ITK-SNAP (version 3.8.0^1^). The ROIs were contoured from both the preoperative and postoperative CTP maps. An attempt was made to draw the same ROIs as the CTP data processing.

### Feature extraction

Feature extraction was performed using RadCloud V.2.2 (Huiying Medical Technology Co., Ltd., Beijing, China). The largest cross-sectional area of the abnormal perfusion was used as the ROI, and the shape-based features were deleted. Of the features, 1,395 were extracted (Supplementary Table 1). The radiomics features were divided into three groups: (a) first-order statistics (126 features), which quantitatively delineated the distribution of voxel intensities within ROIs; (b) textural features (525 features), which were calculated from gray-level run-length matrix, gray-level co-occurrence matrix, gray-level size zone matrix, gray-level dependence matrix, and neighborhood gray-tone difference matrix; (c) wavelet-transformed features (744 features), which included the intensity and texture features derived from wavelet transformation of the original images, processed using filters (e.g., wavelet-LLL, wavelet-LLH, wavelet-LHL, wavelet-LHH, wavelet-HLL, wavelet-HLH, wavelet-HHL, and wavelet-HHH). Delta-radiomics (an example was shown in Supplementary material 1) was defined as the changes in radiomics features pre- and postoperatively during treatment and calculated as follows:


$$\begin{array}{c} \mathrm{Delta}-\mathrm{radiomics}\mathrm{Feature}={\mathrm{Feature}}_{\text{postoperation}}\\ -{\mathrm{Feature}}_{\text{preoperation}}. \end{array}$$

### Radiomic feature reduction and selection

The intraclass correlation coefficient calculated from all the patients was used to improve the reproducibility of the radiomic features (Koo and Li, 2016). Features with intraclass correlation coefficient values > 0.75 were retained for subsequent analysis. All radiomics features were standardized into normal distribution with *z*-score normalization. Then, the independent *t*-test with Bonferroni corrected *p*-values was used to test whether the radiomics features were different between the good and poor collateral formation groups. Finally, the least absolute shrinkage and selection operator (LASSO) algorithm was performed to select the radiomic features. Tenfold cross-validation was used in determining the tuning parameter λ. Some feature coefficients were reduced to zero by tuning the λ. The non-zero coefficient features were selected.

### Radiomics model construction

Two classifiers with machine learning algorithms (support vector machine, SVM; k-nearest neighbors, KNN) were trained for model construction and validated in the training and validation cohorts, respectively. Details of parameters used in machine learning were shown in Supplementary Table 2. The diagnostic performance of the models was evaluated by accuracy, precision, recall, F1 score, and area under the receiver operating characteristic (ROC) curve (AUC) compared with Delong’s test.

### Statistical analysis

Statistical analysis was performed using SPSS statistical software (version 18.0, SPSS Inc., Chicago, IL, United States) and Python software.^2^ Qualitative variables were in *n* (%) and analyzed using Chi-square test, whereas quantitative variables were in mean ± SD and analyzed using *t*-test. Paired *t*-test and two-sample *t*-test were used to analyze differences. ROC curves were constructed to explore the efficacy of CTP parameters and machine learning classifiers. All statistical tests were two-sided, and *p* < 0.05 indicates a significant difference.

## Results

### Patient characteristics

The mean follow-up was 8.2 ± 3.5 months. Fifty-three patients with MMD fulfilled the inclusion and exclusion criteria. The demographic and clinical information of patients are shown in Table 1. All patients were randomly divided into the training (*n* = 42) and test (*n* = 11) cohorts. No significant differences were found in patient characteristics between the training and validation cohorts (Supplementary Table 3).

**TABLE 1:** Clinical information of patients with MMD.

Variables	Patients (*n* = 53)
Age, years*	41.5 ± 12.1(12–62)
**Gender**	
Male	22 (41.5%)
Female	31(58.5%)
**Preoperative clinical symptoms**	
Ischemia	16 (30.2%)
TIA	8 (15.1%)
Infarction	19 (35.8%)
Hemorrhage	10 (18.9%)
**Lesion type**	
Bilateral	40 (75.5%)
Unilateral	13 (24.5%)
Postoperative follow-up, months	8.2 ± 3.5 (3–24)
**Suzuki stages**	
Stage 1	0
Stage 2	1 (1.9%)
Stage 3	26 (49.1%)
Stage 4	24 (45.3%)
Stage 5	2 (3.8%)
Stage 6	0
**Grades for collateral vessel formation after operation**	
Grade 0	2 (3.8%)
Grade 1	15 (28.3%)
Grade 2	26 (49.1%)
Grade 3	10 (18.9%)

TIA, transient ischemic attack.

*Qualitative variables are in *n* (%), whereas quantitative variables are in mean ± SD, with ranges in parentheses.

### Patency of the anastomoses and formation of collateral vessels

**FIGURE 2:**
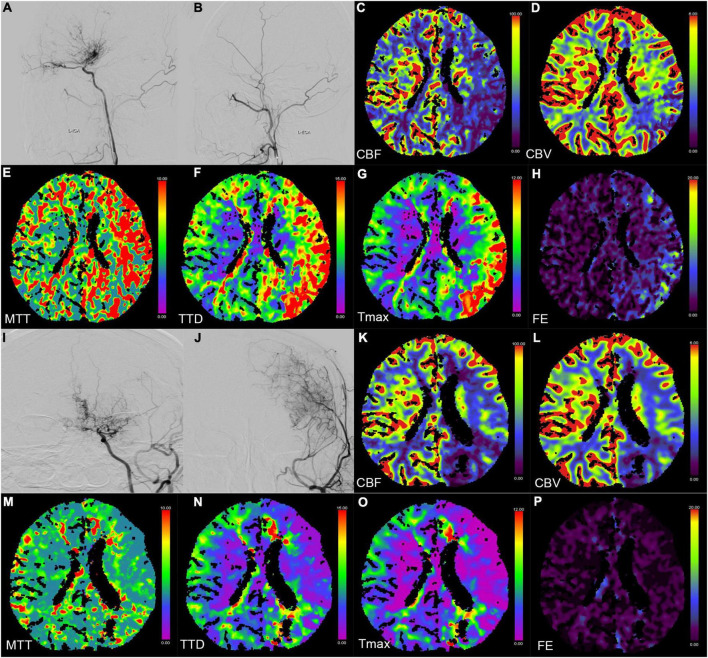
Digital subtraction angiography (DSA) and CT perfusion (CTP) images of a 23-year-old female patient with a history of headache for 2 months (**A–H** preoperation, **I–P** postoperation). **(A,B,I,J)** The DSA images show a patient bypass with supply to the majority of the middle cerebral artery territory after direct bypass surgery. **(C–H,K–P)** The CTP images show that the hemodynamics improved after revascularization in the left hemisphere.

The patency of the anastomoses was evaluated by DSA. Fifty (94.3%) cases showed patency of the bridging vessels (Figure 2), whereas three cases (5.7%) were occluded. Among the 53 patients with collateral vessels formation grade classification, 10 (18.9%), 26 (49.1%), 15 (28.3%), and two (3.8%) cases were grades 3, 2, 1, and 0, respectively. Moreover, 36 and 17 cases were classified into good and poor groups, respectively. Additionally, the patient characteristics of the good and poor groups are presented in Supplementary Table 4.

### CT perfusion evaluations

Six perfusion maps (CBF, CBV, MTT, TTD, Tmax, and FE) were obtained (Table 2 and Figure 2). Compared with preoperative parameters, the results showed that the absolute CBF and rCBF values of the surgical side increased significantly after operation (*p*CBF = 0.023, *p*rCBF < 0.001), whereas the MTT, TTD, Tmax, FE, and their relative values reduced significantly (*p*MTT, *p*TTD, *p*Tmax, *p*rMTT, *p*rTTD, and *p*rTmax < 0.001, *p*FE = 0.005, *p*rFE = 0.002). In addition, no significant difference was noted in CBV and rCBV before and after operation (*p*CBV = 0.351, *p*rCBV = 0.002).

**TABLE 2:** Comparison of CTP values of surgical side before and after operation (mean ± SD).

	Pre-operation	Post-operation	*t*-value	*P*-value
CBF (ml.100 g^–1^.min^–1^)	49.260 ± 21.614	55.671 ± 17.193	–2.337	0.023
CBV (ml.100 g^–1^)	3.595 ± 2.051	3.339 ± 0.923	0.941	0.351
MTT (s)	5.559 ± 1.225	4.381 ± 0.754	6.86	< 0.001
TTD (s)	8.033 ± 2.524	5.523 ± 1.831	7.755	< 0.001
Tmax (s)	5.263 ± 2.077	3.352 ± 1.513	7.293	< 0.001
FE(ml.100 g^–1^.min^–1^)	2.035 ± 1.861	1.181 ± 1.209	2.902	0.005
rCBF	0.848 ± 0.246	0.986 ± 0.206	–4.453	< 0.001
rCBV	1.001 ± 0.227	0.987 ± 0.189	0.451	0.654
rMTT	1.305 ± 0.258	1.028 ± 0.190	7.507	< 0.001
rTTD	1.739 ± 0.806	1.195 ± 0.519	5.840	< 0.001
rTmax	2.215 ± 1.607	1.408 ± 0.904	4.372	< 0.001
rFE	2.385 ± 2.622	1.194 ± 0.746	3.215	0.002

CBF, cerebral blood flow; CBV, cerebral blood volume; TTD, time to drain; MTT, mean transit time; Tmax, time to maximal plasma concentration; FE, flow extraction product.

**FIGURE 3:**
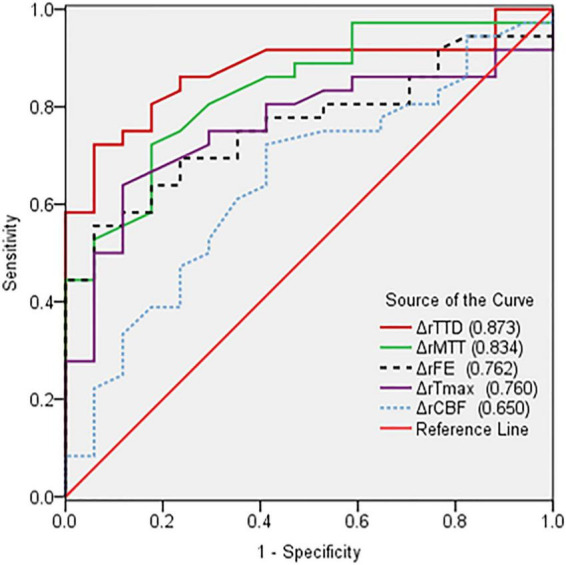
Comparison of receiver operating characteristic curves based on the changes in the relative CTP parameters (ΔrCTP) pre- and postoperation. ΔrTTD had the largest AUC (0.873) among all parameters.

The perfusion improvement between good and poor collateral vessel groups was also compared. The ΔrMTT, ΔrTTD, ΔrTmax, and ΔrFE in the good group were significantly better than those in the poor group (Table 3). However, no statistically significant difference was noted in the ΔrCBF between the two groups. In the ROC curve analysis of the efficacy of the CTP parameters, ΔrTTD had the largest AUC (0.873) among all parameters with a diagnostic sensitivity and specificity of 72.2% and 94.1%, respectively (Figure 3).

**TABLE 3:** Comparison of ΔrCTP values before and after operation between good and poor groups.

	ΔrCBF	ΔrCBV	ΔrMTT	ΔrTTD	ΔrTmax	ΔrFE
Good group(*n* = 36)	0.164 ± 0.238	–0.07 ± 0.229	0.357 ± 0.270	0.823 ± 0.634	1.056 ± 1.554	1.723 ± 3.116
Poor group(*n* = 17)	0.085 ± 0.194	0.105 ± 0.169	0.105 ± 0.163	0.193 ± 0.152	0.281 ± 0.384	0.061 ± 0.633
*t*-value	–1.184	–2.806	–3.556	–5.629	–2.815	–3.070
*p*-value	0.242	0.007	0.001	< 0.001	0.007	0.004

ΔrCTP, the changes in the relative CTP parameters; CBF, cerebral blood flow; CBV, cerebral blood volume; TTD, time to drain; MTT, mean transit time; Tmax, time to maximal plasma concentration; FE, flow extraction product.

### Radiomic feature selection

**FIGURE 4:**
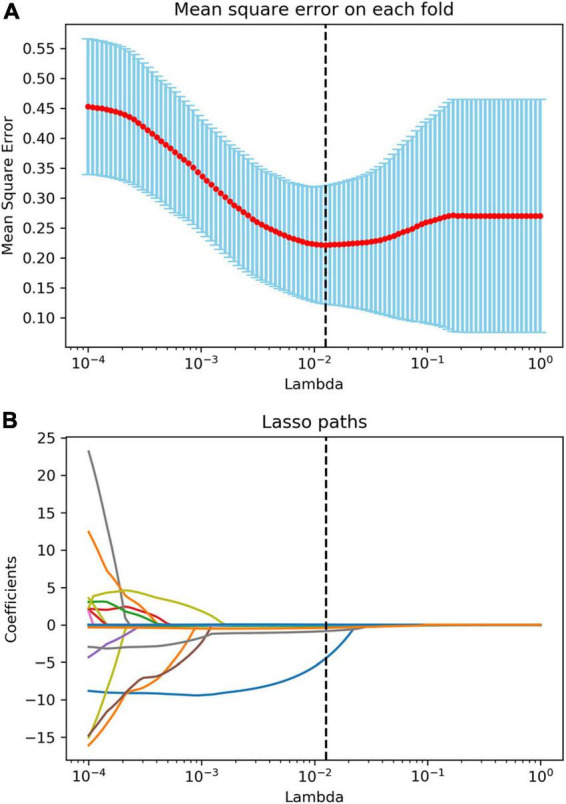
Radiomics feature selection using the least absolute shrinkage and selection operator (LASSO) algorithm. **(A)** The mean square error plot for tenfold cross-validation. The optimal parameter λ (λ = 0.013) in the LASSO algorithm is shown with the smallest mean square error. **(B)** The coefficient profile plot was produced against the log (λ) sequence. At the selected optimal λ value, 11 non-zero coefficients were selected.

**FIGURE 5:**
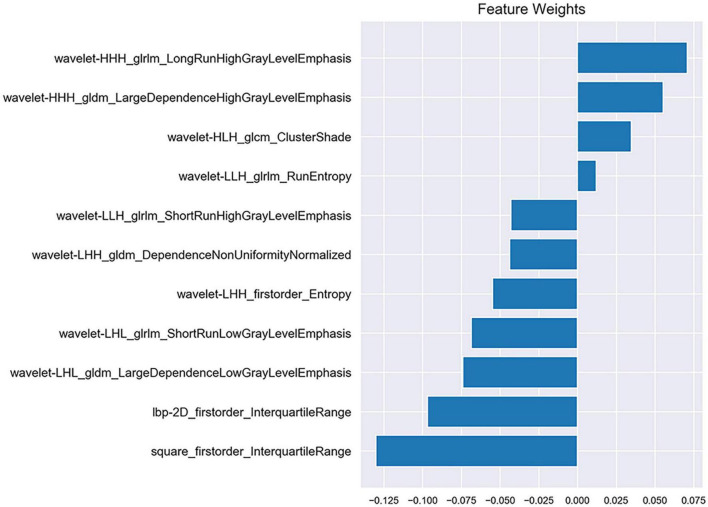
Information of 11 selected features and corresponding feature weights.

After the reproducibility analysis, 1,156 features were left. Using independent *t*-test and LASSO regression model analysis (Figure 4), 11 features were finally left (Figure 5).

### Diagnostic performance of radiomics models

**FIGURE 6:**
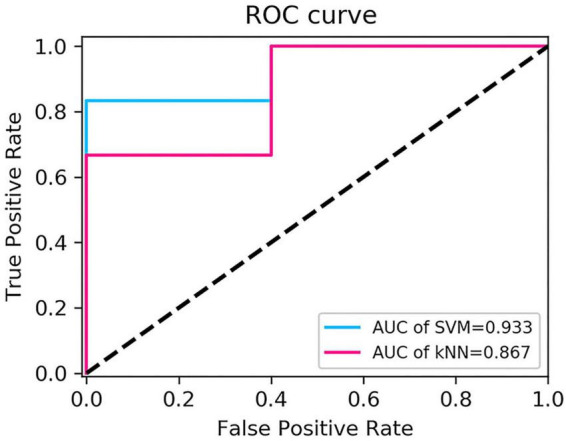
Comparison of receiver operating characteristic curves of the two classifiers.

The performance of the two feature classifiers for the identification of collateral vessel formation is shown in Table 4. The AUC values of SVM and KNN were 0.933 (95% CI, 0.618–0.999) and 0.867 (95% CI, 0.536–0.991), respectively (Figure 6). No significant difference was noted between SVM and KNN classifiers (*p* = 0.394).

**TABLE 4:** Performance of the two feature classifiers for the prediction of collateral vessels formation after revascularization in moyamoya disease.

	Accuracy	Precision	Recall	F1 score	AUC (95% CI)
SVM	0.818	0.750	1.000	0.857	0.933 (0.618–0.999)
KNN	0.636	0.667	0.667	0.667	0.867 (0.536–0.991)

SVM support vector machine, KNN k-nearest neighbors, AUC area under the curve.

## Discussion

Extracranial–intracranial bypass surgery has been widely used in MMD treatment since the first superficial temporal artery-MCA anastomosis was performed by Yasargil in 1967 (Yasargil and Yonekawa, 1977). Traditionally, the surgical outcome was evaluated by DSA. The present study explored whether machine learning could be used to identify collateral vessel formation. Through the analysis of CTP parameters and construction of delta-radiomics models, hemodynamics improvement after revascularization at the surgical side could be evaluated by CTP, and the radiomics models could non-invasively identify collateral vessel formation, where the AUC values of SVM and KNN were 0.933 and 0.867, respectively.

This study adopted the third-generation dual-source CT with DynMulti 4D scan mode. Whole-brain CTP, which can reflect cerebral microcirculation information, was scanned in a single examination with fast scanning speed and low radiation dose (Fang et al., 2016). This study used the Syngo.*via* to process the CTP images. It used the same delay-insensitive deconvolution algorithm with the reference standard CTP software RAPID (iSchema View inc., Menlo Park, CA, United States) (Muehlen et al., 2021). The study found that the rCBF value of the surgical side significantly increased after the operation, while the rMTT, rTTD, rTmax, and rFE values significantly reduced. This finding was consistent with previous research (Zhang et al., 2013; Chen et al., 2016). In the present study, other CTP parameters (e.g., Tmax and FE) were also evaluated. Tmax is the time to the maximum of the tissue residue function. It is an important parameter to differentiate the infarction core from the penumbral tissues (Shih et al., 2003); the optimal threshold for early identification of hypoperfused tissue is between 4 and 6 s (Olivot et al., 2009). The preoperative and postoperative mean Tmax values on the surgical side for total sets in the current study were 5.263 ± 2.077 s and 3.352 ± 1.513 s, respectively (*p* < 0.05). It demonstrated that the ischemic area decreased after revascularization. FE reflects the efflux rate (permeability) from intravenous to extravenous (Roberts et al., 2000), generally regarded as a marker of blood–brain barrier (BBB) disturbance (Xyda et al., 2011). The BBB permeability increases in cerebral ischemia, and the BBB is impaired in MMD patients (Narducci et al., 2019). The absolute and relative values of FE on the surgical side were significantly decreased after revascularization in the current study. This suggested that the BBB was repaired after the revascularization. The parameters through quantitative analysis showed that ischemic cerebral tissue perfusion improved after revascularization. The improvement of cerebral perfusion between the good and the poor groups was further compared after the bypass operation. The postoperative changes in ΔrMTT, ΔrTTD, ΔrTmax, and ΔrFE of the good group were significantly greater than that of the poor group (*p* < 0.05). In addition, this study found that ΔrTTD had the highest efficacy in evaluating the cerebral perfusion improvement, and it may be the most sensitive parameter to evaluate cerebral perfusion. TTD is defined as the time to start + MTT and represents the time in which the contrast agent moves away from the analyzed voxel (Abels et al., 2010; Othman et al., 2016; Vulcu et al., 2019). Thus, it is well-suited to delineate the extent of ischemic lesions.

Radiomics was first proposed by Lambin et al. (2012). Delta-radiomics analysis shows the changes in radiomics features between baseline and follow-up examinations during treatment. This study is believed to be the first to use CTP-based delta-radiomics features to identify collateral vessel formation after revascularization. In the present study, the radiomics changes in MMD before and after revascularization reflected the treatment response. Two machine learning classifiers were trained to identify the formation of collateral vessels. The results showed that the classifiers SVM and KNN had good diagnostic performance with AUC values 0.933 and 0.867, respectively, and no difference was noted between the AUC of SVM and KNN (*p* = 0.394). The algorithms of SVM and KNN are widely used in machine learning (Lee, 2010; Dong et al., 2020). In the present study, the first important radiomics feature was square_firstorder_InterquartileRange. It is a first-order feature and reflects changes in the image array intensity. A previous study found that texture features such as Entropy, uniformity, kurtosis, skewness, and standard deviation of the pixel distribution histogram were correlated with clinical outcomes (Ng et al., 2013). These results implied that the endangered brain tissue was reperfused after vascular anastomosis, and thus led to the intensity and texture change in this area.

The present study had some limitations. The sample size was relatively small. Multicenter prospective studies with a larger set of clinical data are necessary to validate the radiomics model. In addition, the radiomic features extracted in this study were based on two-dimensional (2D) images. Ideally, three-dimensional (3D) image feature extraction should be performed. The study of Lubner et al. (2015) found that the 2D and 3D CT texture results were fairly similar. In addition, there were previous studies used the 2D ROIs to build the radiomics and achieved good performance (Zhou et al., 2019; Arendt et al., 2021).

## Conclusion

CT perfusion could quantitatively access the cerebral hemodynamic changes in MMD before and after revascularization, and TTD maps was the most sensitive parameter in evaluating the cerebral perfusion improvement after revascularization in patients with MMD. The TTD-based delta-radiomics model has the potential to identify collateral vessel formation after the operation, and it may serve as an alternative way to evaluating the MMD revascularization outcomes.

## Data availability statement

The original contributions presented in this study are included in the article/Supplementary material, further inquiries can be directed to the corresponding author.

## Ethics statement

This study was approved by the Ethical Review Committee of the Shandong Provincial Qianfoshan Hospital. Written informed consent for participation was not required for this study in accordance with the national legislation and the institutional requirements.

## Author contributions

JL wrote original draft preparation and design. KL, TJ, DY, ML, and YC collected the data. YZ, CF, JP, and HS analyzed the data and built the prediction models. QZ revised the manuscript. All authors contributed to the article and approved the submitted version.

## Footnotes

1
http://www.itksnap.org

2
https://www.python.org/downloads/release/python-376

## References

[B1] Abels B. Klotz E. Tomandl B. F. Kloska S. P. Lell M. M. (2010). Perfusion CT in acute ischemic stroke: a qualitative and quantitative comparison of deconvolution and maximum slope approach. *Am. J. Neuroradiol.* 31 1690–1698. 10.3174/ajnr.A2151 20581066 PMC7964979

[B2] Acker G. Fekonja L. Vajkoczy P. (2018). Surgical management of moyamoya disease. *Stroke* 49 476–482. 10.1161/STROKEAHA.117.018563 29343587

[B3] Akiyama Y. Mikami T. Mikuni N. (2020). Deep learning-based approach for the diagnosis of moyamoya disease. *J. Stroke Cerebrovasc. Dis.* 29:105322. 10.1016/j.jstrokecerebrovasdis.2020.105322 32992181

[B4] Arendt C. T. Leithner D. Mayerhoefer M. E. Gibbs P. Czerny C. Arnoldner C. (2021). Radiomics of high-resolution computed tomography for the differentiation between cholesteatoma and middle ear inflammation: effects of post-reconstruction methods in a dual-center study. *Eur. Radiol.* 31 4071–4078. 10.1007/s00330-020-07564-4 33277670 PMC8128805

[B5] Bendszus M. Koltzenburg M. Burger R. Warmuth-Metz M. Hofmann E. Solymosi L. (1999). Silent embolism in diagnostic cerebral angiography and neurointerventional procedures: a prospective study. *Lancet* 354 1594–1597. 10.1016/s0140-6736(99)07083-x 10560674

[B6] Chen Y. Xu W. Guo X. Shi Z. Sun Z. Gao L. (2016). CT perfusion assessment of *Moyamoya* syndrome before and after direct revascularization (superficial temporal artery to middle cerebral artery bypass). *Eur. Radiol.* 26 254–261. 10.1007/s00330-015-3802-4 25925360

[B7] Dong F. Li Q. Jiang B. Zhu X. Zeng Q. Huang P. (2020). Differentiation of supratentorial single brain metastasis and glioblastoma by using peri-enhancing oedema region-derived radiomic features and multiple classifiers. *Eur. Radiol.* 30 3015–3022. 10.1007/s00330-019-06460-w 32006166

[B8] Elhalawani H. Lin T. A. Volpe S. Mohamed A. S. R. White A. L. Zafereo J. (2018). Machine learning applications in head and neck radiation oncology: lessons from open-source radiomics challenges. *Front. Oncol.* 8:294. 10.3389/fonc.2018.00294 30175071 PMC6107800

[B9] Fang X. K. Ni Q. Q. Schoepf U. J. Zhou C. S. Chen G. Z. Luo S. (2016). Image quality, radiation dose and diagnostic accuracy of 70 kVp whole brain volumetric CT perfusion imaging: a preliminary study. *Eur. Radiol.* 26 4184–4193. 10.1007/s00330-016-4225-6 26852216

[B10] Gillies R. J. Kinahan P. E. Hricak H. (2016). Radiomics: images are more than pictures, they are data. *Radiology* 278 563–577. 10.1148/radiol.2015151169 26579733 PMC4734157

[B11] Gu D. Hu Y. Ding H. Wei J. Chen K. Liu H. (2019). CT radiomics may predict the grade of pancreatic neuroendocrine tumors: a multicenter study. *Eur. Radiol.* 29 6880–6890. 10.1007/s00330-019-06176-x 31227882

[B12] Guo X. Yuan X. Gao L. Chen Y. Yu H. Chen W. (2021). Encephaloduroarteriosynangiosis (EDAS) treatment of moyamoya syndrome: evaluation by computed tomography perfusion imaging. *Eur. Radiol.* 31 8364–8373. 10.1007/s00330-021-07960-4 33956177

[B13] Huang Y. Q. Liang C. H. He L. Tian J. Liang C. S. Chen X. (2016). Development and validation of a radiomics nomogram for preoperative prediction of lymph node metastasis in colorectal cancer. *J. Clin. Oncol.* 34 2157–2164. 10.1200/JCO.2015.65.9128 27138577

[B14] Hwang I. Cho W. S. Yoo R. E. Kang K. M. Yoo D. H. Yun T. J. (2020). Revascularization evaluation in adult-onset moyamoya disease after bypass surgery: superselective arterial spin labeling perfusion MRI compared with digital subtraction angiography. *Radiology* 297 630–637. 10.1148/radiol.2020201448 32960727

[B15] Kang K. Ma N. Li J. Shen Y. Gu W. Ma G. (2020). Cerebral hemodynamic changes after revascularization in patients with hemorrhagic moyamoya disease. *Front. Neurol.* 11:72. 10.3389/fneur.2020.00072 32117031 PMC7026453

[B16] Kim T. Heo J. Jang D. K. Sunwoo L. Kim J. Lee K. J. (2019). Machine learning for detecting moyamoya disease in plain skull radiography using a convolutional neural network. *EBioMedicine* 40 636–642. 10.1016/j.ebiom.2018.12.043 30598372 PMC6413674

[B17] Kim T. Oh C. W. Bang J. S. Kim J. E. Cho W. S. (2016a). Moyamoya disease: treatment and outcomes. *J. Stroke* 18 21–30. 10.5853/jos.2015.01739 26846757 PMC4747064

[B18] Kim T. Oh C. W. Kwon O. K. Hwang G. Kim J. E. Kang H. S. (2016b). Stroke prevention by direct revascularization for patients with adult-onset moyamoya disease presenting with ischemia. *J. Neurosurg.* 124 1788–1793. 10.3171/2015.6.JNS151105 26636391

[B19] Koo T. K. Li M. Y. (2016). A guideline of selecting and reporting intraclass correlation coefficients for reliability research. *J. Chiropr. Med.* 15 155–163. 10.1016/j.jcm.2016.02.012 27330520 PMC4913118

[B20] Lambin P. Leijenaar R. T. H. Deist T. M. Peerlings J. de Jong E. E. C. van Timmeren J. (2017). Radiomics: the bridge between medical imaging and personalized medicine. *Nat. Rev. Clin. Oncol.* 14 749–762. 10.1038/nrclinonc.2017.141 28975929

[B21] Lambin P. Rios-Velazquez E. Leijenaar R. Carvalho S. van Stiphout R. G. Granton P. (2012). Radiomics: extracting more information from medical images using advanced feature analysis. *Eur. J. Cancer* 48 441–446. 10.1016/j.ejca.2011.11.036 22257792 PMC4533986

[B22] Lee Y. (2010). Support vector machines for classification: a statistical portrait. *Methods Mol. Biol.* 620 347–368. 10.1007/978-1-60761-580-4_11 20652511

[B23] Lei Y. Zhang X. Ni W. Yang H. Su J. B. Xu B. (2021). Recognition of moyamoya disease and its hemorrhagic risk using deep learning algorithms: sourced from retrospective studies. *Neural Regen. Res.* 16 830–835. 10.4103/1673-5374.297085 33229716 PMC8178771

[B24] Li J. Jin M. Sun X. Li J. Liu Y. Xi Y. (2019). Imaging of moyamoya disease and moyamoya syndrome: current status. *J. Comput. Assist. Tomogr.* 43 257–263. 10.1097/RCT.0000000000000834 30589721 PMC6426357

[B25] Lubner M. G. Stabo N. Lubner S. J. del Rio A. M. Song C. Halberg R. B. (2015). CT textural analysis of hepatic metastatic colorectal cancer: pre-treatment tumor heterogeneity correlates with pathology and clinical outcomes. *Abdom. Imaging* 40 2331–2337. 10.1007/s00261-015-0438-4 25968046

[B26] Matsushima T. Inoue T. Suzuki S. O. Fujii K. Fukui M. Hasuo K. (1992). Surgical treatment of moyamoya disease in pediatric patients–comparison between the results of indirect and direct revascularization procedures. *Neurosurgery* 31 401–405. 10.1227/00006123-199209000-00003 1407421

[B27] Miyamoto S. Yoshimoto T. Hashimoto N. Okada Y. Tsuji I. Tominaga T. (2014). Effects of extracranial-intracranial bypass for patients with hemorrhagic moyamoya disease: results of the Japan Adult Moyamoya Trial. *Stroke* 45 1415–1421. 10.1161/STROKEAHA.113.004386 24668203

[B28] Muehlen I. Sprugel M. Hoelter P. Hock S. Knott M. Huttner H. B. (2021). Comparison of two automated computed tomography perfusion applications to predict the final infarct volume after thrombolysis in cerebral infarction 3 recanalization. *Stroke* 53 1657–1664. 10.1161/STROKEAHA.121.035626 34872342

[B29] Narducci A. Yasuyuki K. Onken J. Blecharz K. Vajkoczy P. (2019). In vivo demonstration of blood-brain barrier impairment in Moyamoya disease. *Acta Neurochir.* 161 371–378. 10.1007/s00701-019-03811-w 30675657

[B30] Narisawa A. Fujimura M. Tominaga T. (2009). Efficacy of the revascularization surgery for adult-onset moyamoya disease with the progression of cerebrovascular lesions. *Clin. Neurol. Neurosurg.* 111 123–126. 10.1016/j.clineuro.2008.09.022 18995956

[B31] Ng F. Ganeshan B. Kozarski R. Miles K. A. Goh V. (2013). Assessment of primary colorectal cancer heterogeneity by using whole-tumor texture analysis: contrast-enhanced CT texture as a biomarker of 5-year survival. *Radiology* 266 177–184. 10.1148/radiol.12120254 23151829

[B32] Olivot J. M. Mlynash M. Thijs V. N. Kemp S. Lansberg M. G. Wechsler L. (2009). Optimal Tmax threshold for predicting penumbral tissue in acute stroke. *Stroke* 40 469–475. 10.1161/STROKEAHA.108.526954 19109547 PMC2670783

[B33] Othman A. E. Afat S. Nikoubashman O. Muller M. Schubert G. A. Bier G. (2016). Volume perfusion CT imaging of cerebral vasospasm: diagnostic performance of different perfusion maps. *Neuroradiology* 58 787–792. 10.1007/s00234-016-1695-9 27194077

[B34] Research Committee on the Pathology and Treatment of Spontaneous Occlusion of the Circle of Willis, and Health Labour Sciences Research Grant for Research on Measures for Infractable Diseases (2012). Guidelines for diagnosis and treatment of moyamoya disease (spontaneous occlusion of the circle of Willis). *Neurol. Med. Chir.* 52 245–266. 10.2176/nmc.52.245 22870528

[B35] Roberts H. C. Roberts T. P. Brasch R. C. Dillon W. P. (2000). Quantitative measurement of microvascular permeability in human brain tumors achieved using dynamic contrast-enhanced MR imaging: correlation with histologic grade. *AJNR Am. J. Neuroradiol.* 21 891–899. 10815665 PMC7976746

[B36] Shi Z. Ma G. Zhang D. (2021). Haemodynamic analysis of adult patients with moyamoya disease: CT perfusion and DSA gradings. *Stroke Vasc. Neurol.* 6 41–47. 10.1136/svn-2019-000317 32883875 PMC8005907

[B37] Shih L. C. Saver J. L. Alger J. R. Starkman S. Leary M. C. Vinuela F. (2003). Perfusion-weighted magnetic resonance imaging thresholds identifying core, irreversibly infarcted tissue. *Stroke* 34 1425–1430. 10.1161/01.STR.0000072998.70087.E9 12738899

[B38] Suzuki J. Takaku A. (1969). Cerebrovascular “moyamoya” disease. Disease showing abnormal net-like vessels in base of brain. *Arch. Neurol.* 20 288–299. 10.1001/archneur.1969.00480090076012 5775283

[B39] Vulcu S. Wagner F. Santos A. F. Reitmeir R. Soll N. Schoni D. (2019). Repetitive computed tomography perfusion for detection of cerebral vasospasm-related hypoperfusion in aneurysmal subarachnoid hemorrhage. *World Neurosurg.* 121 e739–e746. 10.1016/j.wneu.2018.09.208 30308346

[B40] Waddle S. L. Juttukonda M. R. Lants S. K. Davis L. T. Chitale R. Fusco M. R. (2020). Classifying intracranial stenosis disease severity from functional MRI data using machine learning. *J. Cereb. Blood Flow Metab.* 40 705–719. 10.1177/0271678X19848098 31068081 PMC7168799

[B41] Xyda A. Haberland U. Klotz E. Bock H. C. Jung K. Knauth M. (2011). Brain volume perfusion CT performed with 128-detector row CT system in patients with cerebral gliomas: a feasibility study. *Eur. Radiol.* 21 1811–1819. 10.1007/s00330-011-2150-2 21573969 PMC3151396

[B42] Yasargil M. G. Yonekawa Y. (1977). Results of microsurgical extra-intracranial arterial bypass in the treatment of cerebral ischemia. *Neurosurgery* 1 22–24. 10.1227/00006123-197707000-00005 615948

[B43] Zaro-Weber O. Fleischer H. Reiblich L. Schuster A. Moeller-Hartmann W. Heiss W. D. (2019). Penumbra detection in acute stroke with perfusion magnetic resonance imaging: validation with (15) O-positron emission tomography. *Ann. Neurol.* 85 875–886. 10.1002/ana.25479 30937950 PMC6593670

[B44] Zhang G. Xu L. Zhao L. Mao L. Li X. Jin Z. (2020). CT-based radiomics to predict the pathological grade of bladder cancer. *Eur. Radiol.* 30 6749–6756. 10.1007/s00330-020-06893-8 32601949

[B45] Zhang J. Wang J. Geng D. Li Y. Song D. Gu Y. (2013). Whole-brain CT perfusion and CT angiography assessment of Moyamoya disease before and after surgical revascularization: preliminary study with 256-slice CT. *PLoS One* 8:e57595. 10.1371/journal.pone.0057595 23451248 PMC3579776

[B46] Zhou J. Tan H. Bai Y. Li J. Lu Q. Chen R. (2019). Evaluating the HER-2 status of breast cancer using mammography radiomics features. *Eur. J. Radiol.* 121:108718. 10.1016/j.ejrad.2019.108718 31711023

