# Supplementary material for: CT perfusion-based delta-radiomics models to identify collateral vessel formation after revascularization in patients with moyamoya disease
Source: Front Neurosci. 2022 Aug 11;16:974096. doi: 10.3389/fnins.2022.974096 (PMC9403315; doi:10.3389/fnins.2022.974096)
Supplement: Supplementary file 1 [file Data_Sheet_1.docx]

**Supplementary Table 1** The 1395 extracted features type extracted from region of interest.

| **Type** | **Meaning of features** | **Numbers** |
| --- | --- | --- |
| First-order | The distribution of voxel intensities within the ROIs. | 126 |
| Textural features | Quantifying regional heterogeneity differences, including gray-level co-occurrence matrix (GLCM), gray-level run-length matrix (GLRLM), gray-level size zone matrix (GLSZM), gray-level dependence matrix (GLDM), and neighboring gray-tone difference matrix (NGTDM). | 525 |
| Wavelet features | Including the intensity and texture features derived from wavelet transformation of the original images, processed using eight filters (wavelet-LLL, wavelet-LLH, wavelet-LHL, wavelet-LHH, wavelet-HLL, wavelet-HLH, wavelet-HHL, and wavelet-HHH). | 744 |

**Supplementary Table 2** Details of parameters used in machine learning.

| **Model** | **parameters** |
| --- | --- |
| SVM | C=0.8, kernel='linear', decision_function_shape='ovr', probability=True |
| KNN | algorithm='auto', leaf_size=30, n_neighbors=8, weights='uniform' |

SVM: Support vector machine, KNN: k-nearest neighbor

**Supplementary Table 3** Patient characteristics in training and validation cohorts.

|  | **Training group (*n* = 42)** | **Validation group (*n* = 11)** | ***χ*^2^/*t* value** | ***p* value** |
| --- | --- | --- | --- | --- |
| Age (years) | 41.9±12.6 | 39.7±10.0 | 0.535 | 0.595 |
| Gender (no. (%)) |  |  |  |  |
| Male | 20(47.6%) | 2(18.2%) | 2.017 | 0.156 |
| Female | 22(52.4%) | 9(81.8%) |  |  |
| Suzuki stages |  |  |  |  |
| Stage 2 | 1(2.4%) | 0 | 0.167 | 0.682 |
| Stage 3 | 21(50%) | 5(45.5%) |  |  |
| Stage 4 | 18(42.9%) | 6(54.5%) |  |  |
| Stage 5 | 2(4.8%) | 0 |  |  |
| Grades for collateral vessel formation after operation | | | | |
| good | 30(71.4%) | 6(54.5%) | 0.497 | 0.481 |
| poor | 12(28.6%) | 5(45.5%) |  |  |
| Mean DSA/CTP follow-up (month) | 7.8±2.8 | 9.2±5.2 | -1.159 | 0.252 |

**Supplementary Table 4** Patient characteristics of the good and poor groups.

|  | **good group (*n* = 36)** | **poor group (*n* = 17)** | ***χ*^2^/*t* value** | ***p* value** |
| --- | --- | --- | --- | --- |
| Age (years) | 40.1±12.6 | 44.4±10.6 | -1.200 | 0.236 |
| Gender (no. (%)) |  |  |  |  |
| Male | 15(41.7%) | 7(41.2%) | 0.001 | 0.973 |
| Female | 21(58.3%) | 10(58.8%) |  |  |
| Suzuki stages |  |  |  |  |
| Stage 2 | 0 | 1(5.9%) | 0.151 | 0.697 |
| Stage 3 | 19(52.8%) | 7(41.2%) |  |  |
| Stage 4 | 15(41.7%) | 9(52.9%) |  |  |
| Stage 5 | 2(5.6%) | 0 |  |  |
| Mean DSA/CTP follow-up (month) | 7.67±2.60 | 9.06±4.74 | -1.385 | 0.172 |
